# Effect of aqueous fraction of *Rosa damascena* on ileum contractile response of guinea pigs

**Published:** 2013

**Authors:** Karim Dolati, Hassan Rakhshandeh, Mohammad Naser Shafei

**Affiliations:** 1 *Pharmacological Research Center of Medicinal Plants and Department of Pharmacology, School of Medicine, Mashhad University of Medical Sciences, Mashhad, I. R. Iran*; 2 *Cognitive sciences Research Center and Department ofPhysiology, School of Medicine, Mashhad University of Medical Sciences, Mashhad, I. R. Iran*

**Keywords:** Aqueous Fraction, Cholinergic System, Guinea Pig, Ileum, *Rosa damascena*

## Abstract

**Objective:** The use of drugs with herbal origin is increasing for treatment of gastrointestinal (GI) disorders. *Rosa damascena (R. damascena) *is a well-known plant suggested to have beneficial effect on GI system*.* In this study, the effect of aqueous fraction of *R. damascena* on the contractions of isolated guinea pig ileum was investigated.

**Materials and Methods**: Aqueous fraction of plant was obtained from ethanolic extract after ethyl acetate and n-butanol fractions were discarded. To evaluate effect of this fraction on ileum contraction, guinea pig ileum was removed and mounted on organ bath and its contraction was recorded. Effect of various concentrations (0.66, 0.83, and 1.3 mg/ml) of aqueous fraction on ileum contraction in comparison with Ach in presence and absence of atropine, a muscarinic antagonist of cholinergic, was evaluated. The response of ileum to 1 µg/ml of acetylcholine was considered as 100% response.

**Results:** Our results showed that aqueous fractions of *R. damascena* dose-dependently increased basal guinea pigs ileum contractions (p<0.05 to p<0.001). Maximal contraction of fraction (1.3 mg/ml) induced 23.4 % of maximal Ach response. The contraction of ileum to aqueous fraction was significant decreased in presence 0.001 µg/ml of atropine.

**Conclusion:** It is concluded that aqueous fraction of *R. damascena* has mild excitatory effect on ileum contraction and this fraction may be beneficial as a mild laxative agent.

## Introduction

In the present time, use of herbal drugs for treatment of several health problems is increased. One of well-known plants that is used in herbal medicine is *Rosa damascena Mill *(*R*. da*mascena*) which has several beneficial effects (Libster, 2002 ▶; Zargari, 1992 ▶; Rakhshandeh et al., 2008 ▶; Hajhashemi et al., 2010 ▶; Boskabady et al., 2011 ▶; Baydar and Baydar, 2013 ▶). 

*R. damascena*, known as Gole Mohammadi in Iran, is popular in the world for its perfume (Loghmani-Khouzani et al., 2007 ▶; Boskabady et al., 2011 ▶). This plant has several therapeutic effects such as treatment of menstrual bleeding, digestive problems, anti-inflammatory, the analgesic, anticonvulsant, antitussive, and bronchodilatory effects (zargari,1992 ▶; Boskabady et al., 2004 ▶; Rakhshandeh et al., 2008 ▶; Hajhashemi et al., 2010 ▶; Shafei et al., 2003 ▶). In addition, we have previously shown that ethanolic and aqueous extract of *R. damascena* has antidepressant effect in rats (Dolati et al., 2012 ▶; Dolati et al., 2011 ▶). The effect of *R. damascena* on digestive system has been shown in traditional medicine. For example, its boiled extract has been used for treatment of constipation (Zargari, 1992 ▶)*.* Recently, effect of *R. damascena* was evaluated in several digestive problems. Abbaszadeh et al. showed that *R. damascena* dose-dependently caused diarrhea in dogs (Abbaszadeh et al., 2010 ▶). 

Similarly, boiled extract gavage of *R. damascena* caused increased frequency of defecation with increased feces water content (Kazerani and Behnam Rassouli, 2011 ▶). In contrast with excitatory effect of *R. damascena *on GI system, its inhibitory effect on digestive system is also reported. For example, intraperitoneal (i.p.) injection of boiled extract has been shown symptoms of constipation (no feces in 24 h) (Kazerani and Behnam Rassouli, 2011 ▶). Inhibitory effect of essential oil of *R. damascena* and its constituents including geraniol and citronellol on rat ileum contraction has been shown (Sadraei et al., 2012 ▶). Furthermore, the rose water (named Golab in Iran) is also used as antispasmodic for treatment of abdominal pain (Mirheydar, 1993 ▶).

Cholinergic system plays an important role in contraction of digestive system (Portbury, 1995 ▶; Guyton and Hall, 2006 ▶) and some gastrointestinal effects are suggested for *R. damascena* mediated by cholinergic system. Several studies have been performed about effect of *R. damascena* on digestive system, but effect of its aqueous fraction on ileum contraction is not evaluated yet. Therefore, in this work, effect of aqueous fraction obtained from flower of *R. damascena* on contraction of guinea pig ileum and its possible effect on cholinergic system were examined.

## Materials and Methods

**Extract** **of plant**

*R. damascena *was collected from Kalate–Nader (an area near Mashhad, east of Iran) and identified by botanists in the Herbarium of the School of Pharmacy, Mashhad University of Medical Sciences Herbarium (No: 254-1804-01).

Flowers of *R. damascena* (200 g) were dried and converted to powder by crushing. The resulting powder was soaked with 1500 ml alcohol 50% and was remained for 72 h at 40 °C inside a heating device. Every day, they were given a shake for 2 to 3 times, after 72 hours, the mixture of rose flower powder and alcohol was cleared and solvent removed by a rotary evaporator. Fifty grams of alcoholic extract was obtained from 200 grams rose flower. The extract for preparation fractions of ethyl acetate, n-butanol, and aqueous fractions was used. In the beginning, 45 g of extract was dissolved in 50 ml distilled water, then mixed with 50 ml ethyl acetate and was poured into the funnel decanter. Soluble material in ethyl acetate was completely isolated from the extract. The remaining extract was thoroughly mixed with 50 ml of the n-butanol and using a funnel decanter, soluble material in n-butanol was also completely isolated from the extract. After removal of ethyl acetate and the n-butanol phase, the remaining of the extract including water-soluble material was poured within plates and put in the bain-marie and with removal of water, the aqueous fraction was obtained.

**Animals**

In this study, 15 guinea pigs (800-1000 g) were used. Animals were obtained from animal room of Mashhad Medical School. Animals   maintained at a temperature of 22-25 °C at 12 h light/dark cycles in appropriate humidity. There were no restrictions for water and diet for the animals. 

Drugs including acetylcholine (Ach) and atropine were purchased from Sigma Company. Ach at concentrations 0.01, 0.03, 0.06, 0.1, and 1 mg/ml alone and in the presence of atropine (0.001 µg/ml) and aqueous fractions of *R. damascena* with concentrations of 0.66, 0.83, and 1.3 mg/ml with and without atropine (0. 001 µg/ml) were used. All drugs were dissolved in saline.

Guinea pigs were sacrificed and their abdomen opened, then the location of ileum was identified, longitudinal strips with 3 cm were separated, gently washed with Tyrode solution, and then transferred into the organ bath. Organ bath was filled with Tyrode's solution and bubbled with a mixture of 95% oxygen with 5% CO_2 _gases. Temperature of water organ bath was maintained at about 37-37.5 °C. Afterwards, tissues were allowed for 15 min to return to stable baseline and recording of ileum contractions was measured by Kymograph. The contractile response of ileum by Ach with concentration of 1 µg/ml (which is equal to 8 cm of contraction) was considered as 100% and other contractions were compared with this response.

**Data** **analysis**

Data were analyzed by SPSS software using one-way ANOVA followed by Tukey-Kramer test. The results were expressed as mean±SEM and considered significant when p<0.05.

## Results

**Effect of** **different concentrations of** **Ach on** **contraction of isolated** **guinea pig ileum** **in absence and presence of atropine**

**Figure 1:**
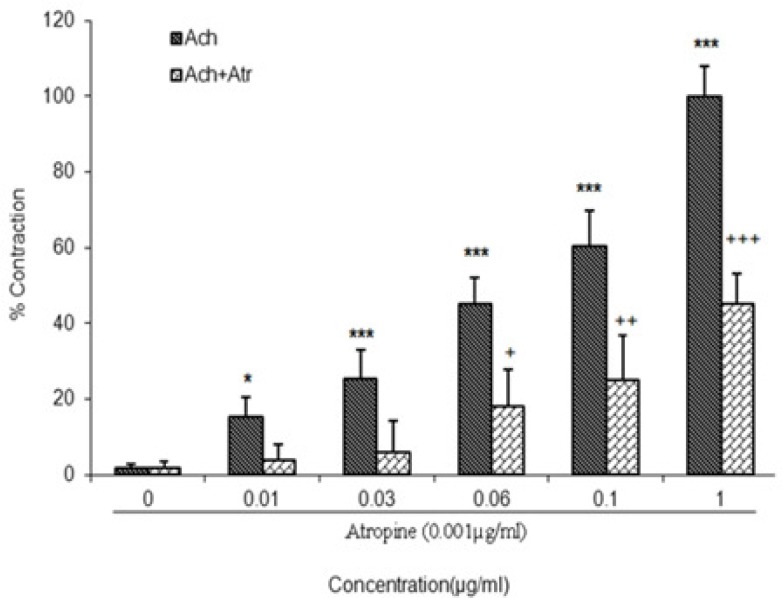
Effect of increasing concentrations of acetylcholine (ACh) on contraction of isolated guinea pigs ileum in absence and presence of atropine . n=7.

Figure 1 indicates the contractile effect of different concentrations of Ach (0.01, 0.03, 0.06, 0. 1, and 1 µg/ml) on isolated guinea pig ileum. As shown, an increase in Ach concentration caused development of contraction and maximum response was seen in concentration of 1 μg/ml. Ileum contraction to Ach was computed based on the percentage and maximal effect induced by concentration of 1 μg/ml which was considered to be 100% response and other responses were compared with that. Contractile effects of various concentrations of Ach on isolated ileum of guinea pig significantly attenuated by atropine (0.001 µg/ml), a muscarinic antagonist receptor (p<.05 to p<0.001; n= 7).

**Effect of** **aqueous** **fractions of** ***R. damascena* on** **contraction of isolated guinea pig ileum**

**Figure 2:**
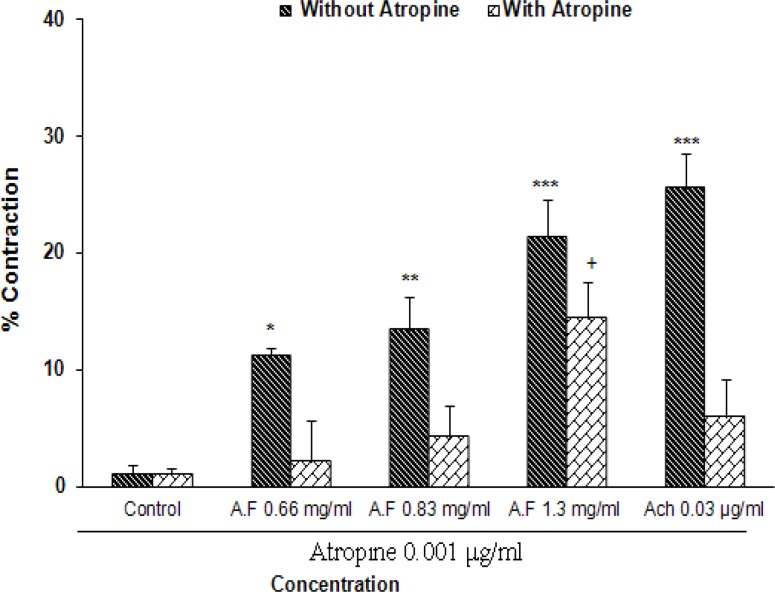
Effect of increasing concentrations of aqueous fraction (AF) of *R. damascena* and Ach (0.03 µg/ml) on contraction of isolated guinea pigs ileum in absence and presence of atropine n=8 Comparison with the control group.

In this experiment, effects of different concentrations of aqueous fraction of *R. damascena* (0.66, 0.83, and 1.3 mg/ml) were examined. Our result showed that this fraction has excitatory effect on ileum contractions. This contractions were started from 0.66 mg/ml and compared with the control significantly increased with additive concentrations (p<0.05 to p<0.001; n=8). Maximum response was achieved in 1.3 mg/ml concentration as shown in Figure 2. Maximal contractile effect of *R. damascena* is comparable with 0.03 µg/ml of Ach.

**Effect of** **aqueous** **fraction of** **R. damascena on contraction of ileum in presence of atropine **

Effect of *R. damascena* on ileum contraction was evaluated in presence of atropine in concentration of 0.001 μg/ml. Our results showed that atropine significantly attenuated contractions elicited by this fraction. However, contraction effect of higher concentration (1.3 mg/ml) was not completely attenuated by atropine and was significantly higher than the control group (p<0.05; Figure 2).

*: Comparison of Ach with the control, *p<0.05, **p<0.01, ***p<0.001

+: Comparison of Ach+Atr with the control, +p<0.05, ++p<0.01, +++ p<0.001.

*p<0.05, **p<0.01, ***p<0.001. +: Comparison of the control group after atropine: +p<0.05.

## Discussion

The results of the present study indicate that the aqueous fraction of *R. damascena* has a significant stimulatory effect on ileum contractions and the peak of its effect is observed in concentration of 1.3 mg/ml.

The contractions of this fraction are comparable with concentration of 0.03 μg/ml of Ach. However, other concentrations of Ach are significantly greater than this effect (Figure 1). These results indicate that aqueous fraction of *R. damascena* has mild excitatory effect on ileum contractions. Our results also show that excitatory effect of aqueous fraction in lower concentrations (0.66 and 0.83 mg/ml) are vigorously blocked in the presence of atropine (Figure 3). However, atropine cannot significantly block higher concentrations of the fraction on ileum contraction. This effect of high dose may be due to the fact that the higher dose of fraction has a toxic effect or it envelopes all of Ach receptors in a way that atropine cannot have any effect on them. In addition, it is possible that the effect of the higher dose of fraction is not merely mediated by cholinergic system and other excitatory neurotransmitters in enteric nervous system such as histamine, serotonin, substance P, and vasoactive intestinal peptide are also involved (Kunze and Furness, 1999 ▶; Guyton and Hall, 2006 ▶; Hansen, 2003 ▶). Our results are consistent with previous studies that point to laxative and prokinetic effects of R. damascena (Abbaszadeh et al., 2010 ▶; Kazerani and Behnam Rassouli, 2011 ▶). However, effect of *R.* *damascena* on gastrointestinal system is controversial and both inhibitory and excitatory effect has been shown. Although several evidences show that excitatory effect of *R.* *damascena* on gastrointestinal system (zargari, 1992 ▶), Sadraei et al. has shown the inhibitory effect of essential oil, geraniol, and citronellol of *R. damascena* on rat ileum contraction (Sadraei et al., 2012 ▶). 

Relaxant effect of aqueous fraction of *R. damascena* on tracheal chain is also reported (Boskabady et al., 2010 ▶). These effects of *R. damascena* maybe are maybe due to the presence of several components in this plant (Loghmani-Khouzani et al., 2007 ▶; Kwon et al., 2009 ▶). Previous studies have identified several ingredients including terpenes, glycosides, flavonoids, anthocyanins, kaempferol, and quarcetin in *R. damascena* (Boskabady et al., 2011 ▶; Baydar and Baydar, 2013 ▶). Each of these components may have different effects on gastrointestinal system. The GI system beside excitatory neurotransmitters also contain inhibitory neurotransmitters such as nitric oxide and opioid receptors (Epstein et al., 1996 ▶; Guyton and Hall, 2006 ▶; Xue et al., 2000 ▶), so, inhibitory effect of this plant is maybe due to the effect on these neurotransmitters. In addition, inhibitory effect of *R. damascena* on ileum contraction or relaxant effect of ethyl acetate on tracheal chains are shown to be induced by compounds such as essential oil, geraniol, citronellol, and ethyl acetate.

All of these fractions are lipid soluble (non-polar), but in our study, excitatory effect on ileum contraction is mediated by aqueous fraction that is soluble in water. Therefore, we suggest that the presence of ingredients that are more soluble in water are involved in excitatory contraction and compounds that are lipid soluble have inhibitory effect on contractions. Future studies are needed to determine ingredients in this fraction and their possible mechanism on ileum contraction. In conclusion, the aqueous fraction of *R. damascena* indicates mild excitatory effect on ileum contraction that is mostly mediated by muscarinic receptor of cholinergic system. Therefore, this fraction may be beneficial as a mild laxative agent.
